# An unusual case of an ACTH-secreting macroadenoma with a germline variant in the aryl hydrocarbon receptor-interacting protein (*AIP*) gene

**DOI:** 10.1530/EDM-14-0105

**Published:** 2015-01-01

**Authors:** Pia T Dinesen, Jakob Dal, Plamena Gabrovska, Mette Gaustadnes, Claus H Gravholt, Karen Stals, Judit Denes, Sylvia L Asa, Márta Korbonits, Jens O L Jørgensen

**Affiliations:** 1 Department of Endocrinology and Internal Medicine, Aarhus University Hospital, Aarhus, Denmark; 2 Department of Molecular Medicine, Aarhus University Hospital, Aarhus, Denmark; 3 Department of Pathology, University Health Network, Toronto, Ontario, Canada; 4 Laboratory Medicine and Pathobiology, University of Toronto, Toronto, Ontario, Canada; 5 Department of Molecular Genetics, Royal Devon and Exeter, Foundation Trust, Exeter, UK; 6 Department of Endocrinology, Bart's and the London School of Medicine, Queen Mary University of London, London, UK

## Abstract

A patient of Cushing's disease (CD) characterized by a large tumor and only subtle symptoms of hormonal hypersecretion was examined. The patient had a germline variant in the aryl hydrocarbon receptor-interacting protein (*AIP*) gene. A 50-year-old male presenting with headache was diagnosed with a large pituitary tumor by magnetic resonance imaging (MRI). His visual fields were intact and he exhibited no features of CD. Owing to an exuberant response to synacthen, an overnight dexamethasone suppression test was performed revealing inadequate suppression of plasma cortisol (419 nmol/l). Owing to tumor growth and visual field impairment, he underwent transsphenoidal surgery and developed hypocortisolemia. The pathology specimen revealed a sparsely granulated corticotrope adenoma. Postoperative MRI showed a large tumor remnant. The patient developed skin hyperpigmentation and a synacthen test demonstrated high basal and stimulated cortisol levels; an overnight dexamethasone suppression test showed no suppression (791 nmol/l) and elevated plasma ACTH levels (135 ng/l). A transcranial operation was performed followed by radiotherapy. Two months after radiotherapy, he developed secondary adrenocortical failure. Genetic testing revealed an *AIP* variant of unknown significance (p.R16H) without loss of the normal *AIP* allele in the tumor. A literature review showed ten CD patients with *AIP* gene variants, of whom five (including our case) were p.R16H. CD is occasionally dominated by pituitary tumor growth rather than symptoms of hypersecretion. The particular *AIP* gene variant identified in our patient is shared by four other reported cases of CD. Future studies are needed to assess whether the reported *AIP* gene variant is more than just coincidental.

**Learning points:**

- CD is occasionally dominated by pituitary tumor growth rather than symptoms of hypersecretion.
- Resolution of both tumor remnant and hormonal hypersecretion may occur within 2 months after postoperative radiotherapy.
- The particular AIP gene variant identified in our patient is shared by four other reported cases of CD.

## Background

Cushing's syndrome refers to the clinical manifestations induced by chronic exposure to excess glucocorticoids. It is a rare condition with an annual incidence of 3/million. It is, however, a frequently entertained differential diagnosis as it shares symptoms and signs with more prevalent conditions, such as type 2 diabetes and polycystic ovary syndrome. In addition, both the diagnosis and treatment of Cushing's syndrome are challenging, which have important prognostic implications. Cushing's disease (CD), defined as pituitary adrenocorticotropic hormone (ACTH) excess, is the most frequent cause of endogenous Cushing's syndrome with an incidence of 2/million per year. It is usually due to a microadenoma (maximal diameter <10 mm) and may be difficult to visualize; in contrast, macroadenomas (maximal diameter >10 mm) comprise only 4–10% of diagnosed patients (1).

The etiology and pathogenesis of corticotrope adenomas remain unclear and the majority appears to be sporadic. In a minority of cases, Cushing's syndrome is associated with genetic syndromes such as McCune–Albright syndrome, MEN1, and Carney complex. More recently, germline mutations in the aryl hydrocarbon receptor-interacting protein (*AIP*) gene have been shown to play a casual role in the development of pituitary adenomas, in particular large somatotropinomas occurring in young patients (2). They have only rarely been recorded to be associated with corticotrope adenomas (2).

This case report involves a male patient incidentally diagnosed with an aggressive pituitary macroadenoma, which proved to be a corticotrope adenoma associated with only subtle features of glucocorticoid excess. Moreover, the patient was diagnosed with a germline variant in the *AIP* gene. In addition to underpinning the broad spectrum of pituitary ACTH excess associated with corticotrope adenomas, we also review published cases of CD associated with *AIP* mutations.

## Case presentation

**Figure 1:**
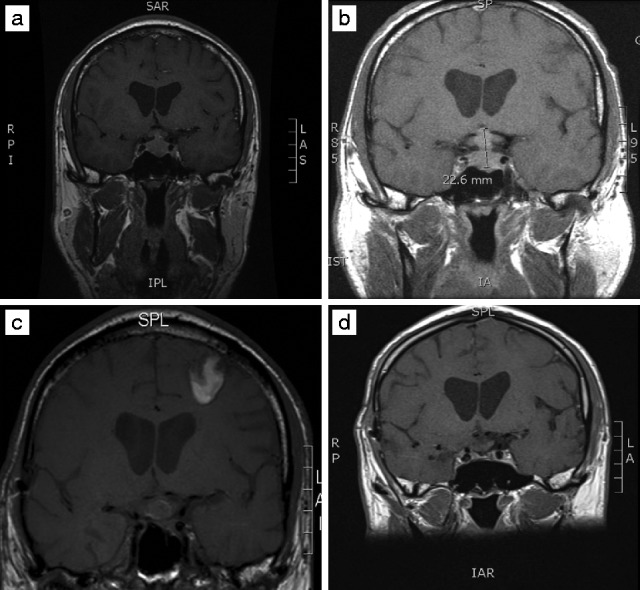
MRI showing T1 post-contrast at the time of diagnosis (a), 7 months after diagnosis (b), 3 months after transcranial surgery (c), and 4 months after conventional radiotherapy (d). Almost 4 years elapsed between the first (a) and the last (d) MRI.

A 50-year-old male was referred to the Department of Endocrinology in Aarhus, Denmark, in January 2007 with a large pituitary tumor diagnosed by magnetic resonance imaging (MRI) performed due to a 1-year history of headaches. Twelve months before this, the patient underwent unilateral orchidectomy for a testicular seminoma and was in remission. In addition, the patient had a history of transitory cerebral ischemia and has been recently diagnosed with type 2 diabetes. The initial MRI revealed a pituitary macroadenoma with suprasellar extension (Fig. 1a). The patient was of normal weight (BMI, 24.8 kg/m^2^) and exhibited no clinical features of anterior pituitary insufficiency, CD or acromegaly, but he did present symptoms of diabetes insipidus with pronounced thirst, polydipsia and polyuria (∼6 l/24 h), also during the night. Iodothyronine and thyroid-stimulating hormone levels were in the low normal range; a short synacthen test showed no evidence of ACTH insufficiency (cortisol levels (nmol/l): 654 (basal) and 981 (30 min)). The patient was started on oral desmopressin, and within 1 month, he was also substituted with levothyroxine and testosterone. A neuro-ophthalmological examination revealed intact visual fields. Owing to a moderately elevated serum insulin-like growth factor 1 (IGF1) level (248 μg/l, ≈2.4 SDS) and the exuberant response to ACTH stimulation, both a growth hormone (GH) profile during an oral glucose load and an overnight dexamethasone suppression test were performed. The former disclosed a near-normal nadir GH level of 0.32 μg/l (<0.30 μg/l), but the patient failed to suppress his morning plasma cortisol (nmol/l) to overnight dexamethasone (648 (afternoon) and 419 (morning)) combined with an unsuppressed ACTH level of 62 ng/l (7–64 ng/l). An MRI performed 7 months after the first one indicated tumor growth (Fig. 1b). Human chorionic gonadotropin and α-fetoprotein levels were normal in the blood and the cerebrospinal fluid.

**Figure 2:**
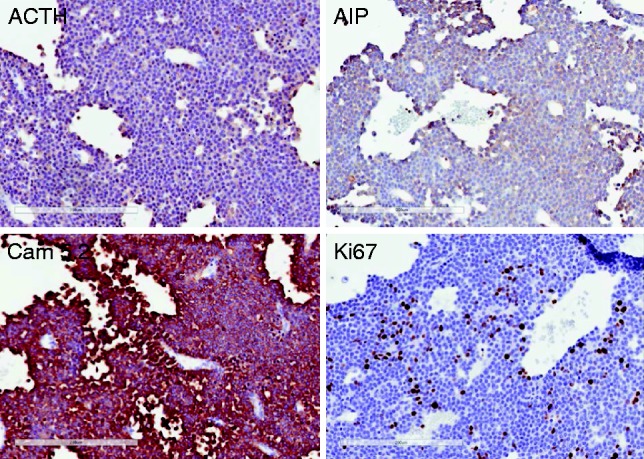
The pituitary tumor exhibits diffuse weak positivity for ACTH and diffuse strong positivity for low-molecular-weight keratins identified by the Cam 5.2 antibody, consistent with a sparsely granulated corticotrope adenoma. AIP immunoreactivity is not lost, but rather shows diffuse staining. The Ki67 labeling index is relatively high for a pituitary tumor at 8–10%.

**Figure 3:**
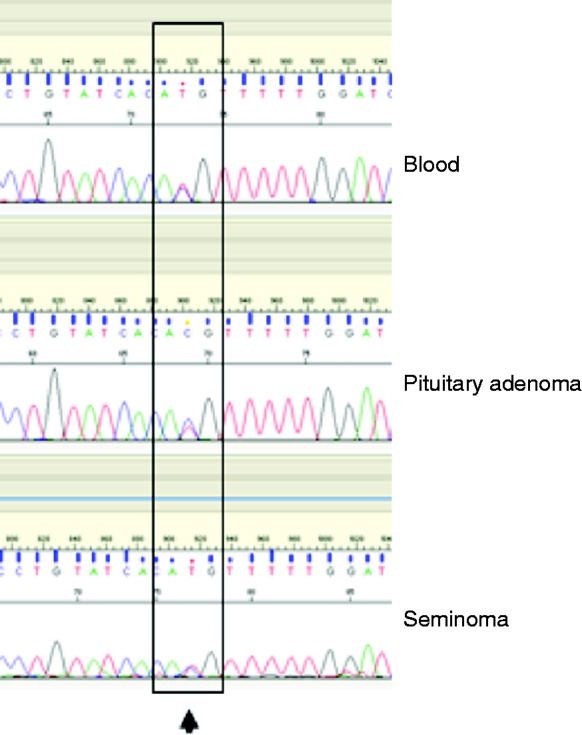
Sequencing results at the c.47G>A; p.R16H locus in germline DNA (blood), pituitary adenoma, and seminoma tissue showing heterozygous status in all three samples, suggesting that the normal allele of the *AIP* gene was not lost in the tumor samples.

The patient underwent transsphenoidal surgery; initial pathology examination identified adenoma tissue but no hormone immunopositivity. Postoperatively, the patient was substituted with oral hydrocortisone due to nausea, fatigue, and a random plasma cortisol level of 61 nmol/l. The patient reported improved vigor after the onset of hydrocortisone replacement and concomitantly had an intended weight loss attributed to rigorous exercise and reduced calorie intake (BMI, 22.5 kg/m^2^). A postoperative GH profile with an oral glucose load showed a slightly elevated nadir GH level (0.37 μg/l) and serum IGF1 level of 248 μg/l (55–220 μg/l). An MRI performed 4 months after the operation demonstrated a large suprasellar tumor remnant; somatostatin analog treatment (Sandostatin LAR 10 mg/4 weeks) was attempted to allow for tumor shrinkage and normalization of GH. A repeat MRI after 6 months showed tumor growth but normal visual function, and treatment with a dopamine agonist (cabergoline 0.5 mg twice weekly) was added. The patient also appeared hyperpigmented and a synacthen test demonstrated high basal (797 nmol/l) and stimulated (1084 nmol/l) cortisol levels. Hydrocortisone treatment was discontinued and a subsequent overnight dexamethasone suppression test again showed lack of suppression (791 nmol/l) and elevated plasma ACTH levels (135 ng/l (reference range: 7–64 ng/l)). His glycemic control deteriorated and insulin treatment was started. The patient was underweight (BMI, 19.0 kg/m^2^). The pathology specimen from the initial operation was re-evaluated and was shown to contain periodic acid-Schiff (PAS) positivity and ACTH immunoreactivity in a pattern consistent with a sparsely granulated corticotrope adenoma (Fig. 2); staining for Pit1 and GH was completely negative and the pattern of keratins decorated with the Cam 5.2 antibody was intense and diffuse in the cytoplasm, consistent with corticotropes rather than somatotropes. Somatostatin analog and dopamine agonist treatment was discontinued and the patient underwent a transcranial operation 2 years after the first operation. In the immediate postoperative phase, the patient developed subarachnoid bleeding and increased intracranial pressure, necessitating lumbar drainage and intubation at the ICU. The patient made a gradual but full recovery and was discharged to his home after 8 days. Hydrocortisone replacement initiated perioperatively was soon discontinued due to elevated plasma cortisol levels. MRI after the transcranial operation revealed a suprasellar tumor remnant (Fig. 1c) and fractionated conventional radiotherapy was initiated 3 months postoperatively. This was complicated by severe headache treated with oral prednisolone, and also a generalized seizure, which was attributed to a small infarction of the right temporal lobe. The patient was started on oral lamotrigine. A synacthen test performed 2 months after discontinuation of prednisolone treatment indicated secondary adrenocortical failure and the patient resumed hydrocortisone replacement. MRI performed ∼4 months after completion of radiotherapy showed resolution of the suprasellar tumor remnant (Fig. 1d). Insulin treatment was discontinued 10 months after radiotherapy. A GH stimulation test documented GH deficiency and low-dose GH replacement was started. The patient underwent genetic testing for *AIP* mutation and a variant of unknown significance was identified (p.R16H). The patient had no family history of pituitary disease. The seminoma and the pituitary adenoma tissue did not show any loss of the normal *AIP* allele (Fig. 3). The patient is presently doing well although retired. His medication includes hydrocortisone, levothyroxine, testosterone, GH, and desmopressin. He also continues lamotrigine treatment at a reduced dose but does not require medical treatment for diabetes mellitus with a BMI of 23.2 kg/m^2^.

## Investigation

Overnight dexamethasone suppression rest revealed elevated cortisol (791 nmol/l) and ACTH levels (135 ng/l). Immunohistochemistry showed PAS positivity and ACTH immunoreactivity in a pattern consistent with a sparsely granulated corticotrope adenoma. Genetic testing indicated the *AIP* variant p.R16H without loss of the normal *AIP* allele in the pituitary adenoma tissue.

## Treatment

MRI after the transcranial operation revealed a suprasellar tumor remnant and fractionated conventional radiotherapy was initiated 3 months postoperatively.

## Outcome and follow-up

A synacthen test was performed 2 months after the conventional radiotherapy and revealed secondary adrenocortical failure. The patient developed permanent panhypopituitarism and is currently treated with hydrocortisone, levothyroxine, testosterone, GH, and desmopressin.

## Discussion

This case of CD is educational in several respects. The presenting symptom was headache, which led to an MRI revealing a pituitary tumor. It is important to recognize the spectrum of pituitary ACTH excess associated with corticotrope adenomas. The majority of patients with CD are diagnosed on the basis of classic symptoms and signs of hypercortisolism; the traditional challenge in the context of CD is rather the opposite than in our case: a patient presenting with persuasive symptoms and signs but without a distinct pituitary tumor. Most ACTH-producing pituitary adenomas are microadenomas (<10 mm in diameter); these small tumors are usually composed of basophilic, densely granulated corticotropes. In contrast, a less frequent scenario is that of a macroadenoma associated with only subtle features of glucocorticoid excess; these ‘whispering’ adenomas, distinct from the truly silent corticotrope adenomas, are almost always composed of chromophobic cells with faint immunoreactivity for ACTH that can be easily missed (3). Awareness of the clinicopathological features of these corticotrope adenoma variants is important in patient management. A simple approach is ‘big Cushing, small tumor; big tumor, small Cushing’.

Our patient is unusual in that his pituitary adenoma caused central diabetes insipidus, which is usually attributed to other mass lesions involving the pituitary stalk or occurs following pituitary surgery.

The initial endocrine evaluation included an elevated serum IGF1 measurement and a subsequent GH profile following an oral glucose load demonstrated a slightly elevated nadir GH level indicative of acromegaly despite the absence of classical symptoms and signs. Only few cases of pituitary tumors secreting both GH and ACTH are reported; in our case, the tumor tissue submitted for pathology examination was completely negative for Pit1 and GH and had no features of a somatotrope adenoma. We therefore find it unlikely that the tumor co-secreted GH, and it should be noted that elevated serum IGF1 levels have previously been reported in Cushing's syndrome (CS) (4).

Given the fact that the patient had an ACTH-producing adenoma and only a small proportion of the tumor was removed, it was also unexpected that hypocortisolemia developed after the first operation. The rapid resolution (4 months) of hypercortisolemia following the conventional radiation therapy targeting the suprasellar tumor remnant, and development of secondary adrenocortical failure are also unusual, as the effects of conventional radiation therapy on hypersecretion and tumor shrinkage usually set in after several years. We suggest that an infarction developed in the pituitary tumor remnant, which, to our knowledge, has been reported in only one previous case – also a patient with CD – following radiation therapy (5).

Finally, our case was diagnosed with a variant in the *AIP* gene c.47G>A, p.R16H. *AIP* mutations are associated with familial pituitary adenomas and more than 200 cases have been described displaying over 70 different mutations (2). The pituitary adenomas with *AIP* mutations are predominantly GH-producing macroadenomas. Thus far, ten cases of CD have been reported (6) (7) (8) (Table 1). The particular *AIP* variant reported in our case is considered of unclear pathological significance. It has been identified in 0.3% of European Americans and 0.08% of African Americans in the general population. An *in vitro* functional study based on binding to one of the partners of AIP phosphodiesterase subtype 4A5 did not demonstrate a significant functional change attributable to this gene variant. Nevertheless, this gene variant has been reported in four additional patients with CD (Table 1). In accordance with the Knudson two-hit hypothesis, loss of heterozygosity (LOH) at the *AIP* gene has been detected in the majority of somatotropinomas with germline mutation (2). However, in our case, the pituitary tumor tissue lacked LOH, and AIP was detected by immunohistochemistry. LOH status has been reported in one additional case with CD (Table 1, patient no. 10), which also revealed lack of LOH. With lack of loss of the second *AIP* allele, a dominant negative mechanism could theoretically explain the role of AIP in these corticotrope adenomas. However, as loss of the glucocorticoid receptor (GR) has been linked to CD (9), and as AIP is known to inhibit the activity of GR (10), if R16H leads to loss of AIP function, increased GR activity would be predicted, which in turn would enhance glucocorticoid feedback inhibition and therefore suppress adenoma formation. It has been recently shown that, in addition to the C-terminal TPR domain, the N-terminal PPI domain of AIP can also bind Hsp90 (11). As Hsp90 is part of the GR complex, sequence alterations in the PPI domain (amino acids 13–160), such as the R16H variant, might influence AIP–Hsp90 binding and the function of Hsp90 and therefore the GR complex. It remains yet to be investigated whether the p.R16H gene variant may have an impact on GR function.

**Table 1:** Patients with ACTH-secreting adenomas and variants identified in the *AIP* gene. A compilation of reported cases of Cushing's disease with variants in the *AIP* gene. Data on sex, age, and tumor size are lacking in some cases (−)

**Patient no.**	**Sequence change**	**Variant type**	**Sex** (M/F)	**Age at diagnosis**	**Size** (mm)	**Country of origin**	**Reference**
1	c.47G>A, p.R16H	Missense	M	50	Macro	Denmark	Current report
2	c.47G>A, p.R16H	Missense	−	−	−	Poland	(8)
3	c.47G>A, p.R16H	Missense	−	−	−	Poland	(8)
4	c.47G>A, p.R16H	Missense	−	−	−	Poland	(8)
5	c.47G>A, p.R16H	Missense	M	14	Micro	France	(7)
6	c.911G>A, p.R304Q	Missense	−	26	−	Poland	(8)
7	c.696G>C, p.P232P	Missense	−	−	−	Poland	(8)
8	c.308A>G, p.K103R	Missense	M	6	Micro	USA	(6)
9	c.26G>A, p.R9Q	Missense	F	39	Micro	France	(7)
10	c.752delT, p.L251RfsX52	Frameshift	F	25	Macro	France	(7)

## Patient consent

Written informed consent was obtained from the patient.

## Author contribution statement

P T Dinesen and J Dal were involved in writing the case report and data collection. P Gabrovska, K Stals, and J Denes performed genetic examination of the pituitary adenoma tissue. M Gaustadnes and C H Gravholt performed genetic testing for *AIP* mutation. S L Asa was responsible for pituitary tumor histology, the histological perspective of the case rapport. M Korbonits was responsible for the genetic perspective of the case rapport. J O L Jørgensen was in charge of the patient treatment and the CD perspective of case rapport.
